# Role of mTORC1 signaling during memory consolidation in Alzheimer’s disease

**DOI:** 10.1002/alz.090773

**Published:** 2025-01-03

**Authors:** Magdalena Pereyra, Christoph Schmidt‐Hieber, Brice Bathellier

**Affiliations:** ^1^ Institut de l'Audition/Institut Pasteur, Paris France; ^2^ Friedrich Schiller University Jena, Jena Germany

## Abstract

**Background:**

Memory consolidation is an essential process for our everyday lives that is severely disrupted in Alzheimer’s Disease (AD). Memories are initially encoded in the hippocampus before being consolidated in the neocortex by synaptic plasticity processes that depend on protein synthesis. However, how molecular pathways affect synaptic signalling during memory consolidation in health and disease is unclear. We hypothesize that mechanistic Target Of Rapamycin Complex 1 (mTORC1), a central regulator of protein synthesis, plays an essential role in the communication between the hippocampus and the prefrontal cortex (PFC), and that mTORC1 dysregulation contributes to memory dysfunction in AD.

**Method:**

We evaluated the role of mTORC1 in memory consolidation using a PFC‐dependent spatial object recognition (SOR) task. We used a mouse model of AD where mice express a human variant of the amyloid precursor protein (hAPP). We extend our results to another amyloid‐based mouse model (5XFAD 6,5 months old). Furthermore, we have evaluated mTORC1 pathway‐associated protein levels by Western Blot in both AD mice models.

**Result:**

First, we found that infusion of rapamycin, a selective mTORC1 inhibitor, into the PFC of WT animals immediately but not 3 hours after training disrupted long‐term memory expression.

We observed that hAPP‐injected animals exhibited impaired long‐term memory expression in the SOR task (tested 6 weeks after hAPP injections in PFC) as well in the novel object recognition task compared to control mice. We evaluated mTORC1 pathway‐associated protein levels by Western Blot in hAPP‐expressing or control mice (1,5 months after viral injections) and found no differences in PFC or hippocampal samples. We repeated biochemical measurements in untrained hAPP or control mice 4 months after injections and found a trend towards downregulation of mTORC1 pathway‐associated protein levels in PFC.

Finally we also observed great memory retention deficits in 5XFAD mice(6,5 months old) as well as a significant downregulation of mTORC1 pathway‐associated protein levels in PFC and hippocampal samples.

**Conclusion:**

Our preliminary data suggest a downregulation of mTORC1 activation in two different AD mice models. Taken together, our results point to a key role of mTORC1 during memory consolidation in PFC that might be disrupted during AD.

